# Making Diabetes Care Fit—Are We Making Progress?

**DOI:** 10.3389/fcdhc.2021.658817

**Published:** 2021-04-16

**Authors:** Merel M. Ruissen, René Rodriguez-Gutierrez, Victor M. Montori, Marleen Kunneman

**Affiliations:** ^1^ Division of Endocrinology, Department of Medicine, Leiden University Medical Center, Leiden, Netherlands; ^2^ Knowledge and Evaluation Research Unit, Mayo Clinic, Rochester, MN, United States; ^3^ Plataforma INVEST Medicina-UANL—KER Unit, KER Unit México, Universidad Autónoma de Nuevo León, Monterrey, Mexico; ^4^ Endocrinology Division, University Hospital “Dr José E González,” Monterrey, Mexico; ^5^ Division of Endocrinology, Diabetes, Metabolism and Nutrition, Mayo Clinic, Rochester, MN, United States; ^6^ Division of Medical Decision Making, Department of Biomedical Data Sciences, Leiden University Medical Center, Leiden, Netherlands

**Keywords:** patient involvement, patient-centered care, shared decision making, minimal disruptive care, diabetes mellitus, fit

## Abstract

The care of patients with diabetes requires plans of care that make intellectual, practical, and emotional sense to patients. For these plans to fit well, patients and clinicians must work together to develop a common understanding of the patient’s problematic human situation and co-create a plan of care that responds well to it. This process, which starts at the point of care, needs to continue at the point of life. There, patients work to fit the demands of their care plan along with the demands placed by their lives and loves. Thought in this way, diabetes care goes beyond the control of metabolic parameters and the achievement of glycemic control targets. Instead, it is a highly individualized endeavor that must arrive at a care plan that reflects the biology and biography of the patient, the best available research evidence, and the priorities and values of the patient and her community. It must also be feasible within the life of the patient, minimally disrupting those aspects of the patient life that are treasured and justify the pursuit of care in the first place. Patient-centered methods such as shared decision making and minimally disruptive medicine have joined technological advances, patient empowerment, self-management support, and expert patient communities to advance the fit of diabetes care both at the point of care and at the point of life.

## Introduction

Diabetes care should improve the health-related quality of life of patients with diabetes, both type 1 and type 2 diabetes, and mitigate their risk, morbidity, and mortality from chronic micro- and macrovascular complications. As with any other chronic condition, it is patients and their caregivers who must implement diabetes care plans. These plans should respond to the patient’s problematic situation in at least three ways (1). First, they must be scientifically sound, addressing what is known about the determinants of outcomes with evidence-based interventions, favoring interventions that respond well to the situation of the patient and promote outcomes that the patient values. Second, their implementation must be feasible in their daily routines and should disrupt those routines to the least extent possible. Third, patients must feel that the plan is the right thing to do for them at the present time. Plans that make intellectual, practical, emotional sense to patients are said to configure “care that fits.” Patients with plans of care that do not fit, “receive tests and treatments they do not need, understand or implement, a result that is wasteful and harmful” (2).

In this perspective, we consider how to make diabetes care fit, the role of patients and clinicians within this process, and potential strategies to do so. A distinction can be made between making care fit at the *point of care* and at the *point of life.* Fitting at the point of care occurs mostly during clinical encounters and demands collaboration between patients and clinicians, a term we use capaciously to include physicians, therapists, pharmacists, nurses, and any other professionals with the privilege of directly participating in the patient’s care (3). In designing care plans, patients and clinicians must work together to uncover the problematic situation of the patient and to determine how to best respond to it. At the point of life, patients and caregivers must integrate daily the practical demands of the care plan (and, often, of navigating health care) with other demands of life and living. The patient is usually the one person bridging these two efforts to make care fit.

## Fitting at the Point of Care

The process of making care fit requires attention to the biology and the biography of the patient, to their physiology and their psychology. Care must respond to the problematic biological situation of the patient, be evidence-based, and make sense given a patient’s unique context. Within the process of fitting care clinicians should also assess the patient’s capacity to carry the burden of disease and treatment and potential diabetes-related distress, in order to prevent overburdening the patient. In doing so, some barriers emerge, such as restrictions in the individualization of treatment plans and the practical difficulties patients must face when implementing treatments for diabetes and its comorbidities.

In addition to smoking cessation, lipid and blood-pressure control, and aspirin use, intensive glycemic control (i.e., HbA1c ≤7%) is recommended to achieve diabetes care goals (4, 5). And yet, when compared to conventional glycemic control (HbA1c <8.0–8.5%), intensive glycemic control has not consistently reduced the risk of complications of type 2 diabetes, while it has increased the cost and complexity of treatment and the risk for hypoglycemia (6–9). Furthermore, a narrow focus on glycemic control may fail to consider the biological (e.g., comorbidities) and biographical (e.g., values, preferences and financial, family, and social) facets of patients’ problematic situations. Guidelines with fixed HbA1c targets and formularies that limit the range and order (i.e., use of first-line agents followed by second-line agents) of the diabetes medications may excessively constrain the possibility of making care fit through treatment individualization.

Severe and long-standing type 2 diabetes often requires the use of multiple medications to achieve glycemic control. Fitting care must consider not just the efficacy of each of these drug regimens but their additive and interacting effects in terms of inconveniences, costs, and harms (10). To illustrate this cumulative burden of treatment, consider how antihypertensives, introduced to reduce cardiovascular risk associated with comorbid hypertension, can cause orthostatic dizziness which may compound dizziness caused by gabapentin introduced to treat painful neuropathy. This treatment burden extends beyond the effects of polypharmacy to also include the demands health care makes on patients in terms of time, energy, and attention given how health care is organized and delivered. Together, the healthcare workload that patients must shoulder lead to reductions in quality of life, a phenomenon sometimes called the burden of treatment (11). These burdens can be tolerated better when they are clearly connected with advancing patient goals and priorities while efforts are put in place to minimally disrupt patient lives (12). The latest guidelines by the American Diabetes Association recognize the need to align diabetes treatment with patient goals, by recommending flexible treatment goals and programs and by recognizing burden of treatment as a key consideration.

## Patient Empowerment and Patient-Centered Care

Empowered patients choose personal, meaningful, and realistic goals for care, think critically, act autonomously, and enhance their self-efficacy (13, 14). Empowered patients would therefore be in a better position to take part in fitting of care at both the point of care and at the point of life, where treatment strategies and associated tasks must be carefully woven into that patient’s daily routine. Interventions to promote patient empowerment, such as self-management education programs, have shown some benefits in the self-management of patients with diabetes, but their quality is inconsistent, and access remains patchy (15). These programs, however, often seek to improve patients’ adherence to recommended care, rather than to increase patient’s autonomy and fitness of care (14). This may be frustrating to both patients and clinicians, with patients feeling they are failing and clinicians labeling patients as non-compliant and blaming them for not meeting recommended targets (14). To make care fit, empowerment must not contribute to this conflict but rather promote patient-clinician collaboration. Patient-centered care describes such a collaboration.

Patient-centered care is “respectful of and responsive to individual preferences, needs, and values” (16) through effective communication, partnership, and health promotion (17). Using effective communication, clinicians gain insight into the patients’ personal situation, their experiences, their priorities, goals, preferences, and values. This enables patients and clinicians to form a partnership, an alliance to find the treatment plan that best fits the patient’s personal situation, and effectively promotes health (18). Furthermore, clinicians need to sufficiently inform patients about potential treatment options and provide them with the opportunity to take advantage of all available resources (19). Patient-centered care is therefore an approach to co-produce sensible plans of care that can feasibly contribute to desirable patient outcomes (2).

## Shared Decision Making in Diabetes Care

A person-centered approach to design care plans that fit is shared decision making (SDM) (1, 20). SDM is a conversation in which patients and clinicians develop a common view of the patient’s situation and co-create a plan of care to address it. When successful, SDM leads to a care plan that is more likely to *respond well* to the problematic situation of the patient, to be *feasible* given the existing demands on the patient’s time, energy, and attention, and to be *desirable* given the patient’s expectations, preferences, goals, and values. In this way, SDM contributes to make sure the plan of care makes intellectual, emotional, and practical sense and therefore fits well within the patient’s life (21). In the care of patients with diabetes and other chronic conditions, patients contribute to SDM as experts in the impact of disease and treatment in their personal context (22); over time, patients develop expertise on what is feasible and what not in their unique situation (23). This information and expertise contributes to the biomedical evidence and clinician experience as these partners share the decision-making process.

Since SDM is a method of care, clinicians and patients can determine when and how to engage in this method and whether or not to use supportive tools. Although implementation science is exploring how best to enact shared decision making in practice, its application depends more on how well it can address the patient’s problem than on the type of encounter (new or continuity, in-person or remote), the type of clinician, or on the availability of tools.

Interventions to facilitate SDM have been shown to improve patient (risk) knowledge and decisional comfort (24). Importantly, most patients prefer to participate in decision making, when they get the information and knowledge that they need to make decisions (25). As a result, healthcare policies and guidelines recommend SDM and the use of SDM tools (26). These aids can be designed to support the SDM conversation and often offer the necessary information in a useful and accessible form for use in preparation for or during the consultation (25). Tools designed for use during the consultation may be particularly effective in guiding patients and clinicians through the shared decision-making process, such as fostering of choice awareness, discussing the available options, exploring patient priorities, and making a final decision (27, 28). These tools can be easily used by all types of clinicians without substantial training to enable effective and efficient communication and guide the decision-making process to reflect the patients’ situation, preferences, and needs.

Consider the case of a patient with type 2 diabetes and without complications who has implemented a healthy lifestyle and uses metformin without achieving her glycemic target. Her clinician may select a second-line option according to recommended algorithms based on her degree of hyperglycemia and her cardiovascular and renal state. This medication may very well be the right one or may be too burdensome to this patient because of its side effects, complexity of administration, or out-of-pocket costs. Alternatively, her clinician could engage the patient in SDM, even using a validated SDM tool, such as the *Diabetes Medication Choice* decision aid (29), and improve the likelihood that the medication selected would fit better.

## Minimally Disruptive Medicine

Because of how health care is organized and delivered, it often delegates medical errands to patients, transferring navigational and administrative tasks that worsen the burden of treatment and disrupt their lives and the lives of their caregivers. The multiple, often uncoordinated, care paths involved in the care of diabetes and of its associated comorbidities, together with the physical and emotional burden of disease for both the patient and their family may be overwhelming to patients and caregivers with consequent decay in the fidelity with which treatments are implemented at the point of life. When co-creating patient-centered plans of care, patients and clinicians pursue patient priorities for health care while minimizing the burden of treatment. Using this approach, sometimes called minimally disruptive medicine (MDM) (12), health care seeks the most effective yet least burdensome treatment for each patient in particular while reducing, in general, the tasks delegated to patients and caregivers. MDM is patient focused to the extent that it respects patients’ limited time, energy, and attention, while accounting for patients’ usual prioritization of these precious resources to attend the demands their lives and loves place over those placed by the need to complete healthcare tasks when the latter conflict with the former.

In diabetes, MDM is particularly important when patients have to implement complicated treatment regimens: estimate and administer insulin doses, monitor glucose levels, implement an accurate accounting of carbohydrates and caloric intake, and so on. MDM is also critical when patients must implement the care of each of their existing conditions which, in addition to diabetes-specific tasks, often contributes to polypharmacy and complex dietary restrictions. This can easily and frequently lead to an unsustainable burden of treatment which may lead to non-adherence. Mindful of the burden of treatment, clinicians and patients may co-create plans of care that fit the particular needs of the patient in a manner that renders them feasible within their daily routines. Diabetes technologies such as continuous glucose monitors (CGM) (30), continuous subcutaneous insulin infusion (CSII) devices, and automated insulin dosing (AID) systems (31) are now available for the care of patients with type 1 diabetes and severe type 2 diabetes. These systems can contribute to reduce glucose variability and improve glycemic control without a large increase in the risk of hypoglycemia. For patients for whom technology adoption is relatively easy, who can afford these devices, and who can access parts and services with minimal friction and cost, the adoption of these technologies may translate into care with a reduced burden of treatment (32, 33). However, for others these technological advances may increase the burden of disease, time, and treatment and may result in an increase in diabetes distress (34). These digital advances may therefore cause a divide between patients that benefit from new technology, both biomedically and psychologically, and those for whom these technological advances result in an increased burden and associated diabetes distress.

Some MDM tools, such as the Instrument for Patient Capacity Assessment (I-CAN) (35), exist to make care fit during the consultation by facilitating conversations about treatment burden. This tool supports patients and clinicians in assessing if and how a certain healthcare strategy may interact with the patient’s life, and in clarifying how aspects of the patient’s life may interact with the treatment plan (36). Most MDM tools (like I-CAN) can be easily used during the consultation by any clinician without additional training.

## Fitting at the Point of Life: The Ongoing Work of Being a Patient

Patients and clinicians can work together to make care fit at the point of care, that is, in clinical encounters, but patients will face an ongoing process of fitting care in their personal life. It is at the point of life where some care plans prove feasible or infeasible. Yet, this process, which occupies the vast majority of the time persons experience as patients, is often invisible to clinicians. Advances in diabetes technology and patient communities are contributing to the fitness of care at the point of life.

### New Technologies to Support Patients With Diabetes

To support successful implementation of care plans, the last decade has shown an emergence of new self-management technologies, such as smart insulin pens (37), insulin pumps (33), and closed-loop systems (38). Furthermore, glucose monitoring has evolved to include flash glucose sensors (39–41), continuous glucose monitors (30), and e-health systems that support the patient in their diabetes self-management. While able to improve glycemic control and reduce the risk of hypoglycemia, the extent to which these technologies contribute to or reduce diabetes chronic complications and burden of treatment remains uncertain. Technologies such as CGM and CSII can improve patient satisfaction and acceptability, and reduce diabetes worries and interpersonal hassles for some patients (42). This is important as the use of this devices may probably translate into a reduced burden of treatment that can make diabetes treatment more bearable for people with diabetes, particularly those living with type 1 diabetes. However, for some patients the time and efforts needed to adopt these technological advances and the associated focus on glycemic control may negatively impact the burden of disease and treatment. Furthermore, these new technologies require connection to online platforms or applications and data sharing agreements with third parties. Thus, they contribute to patient work by demanding that patients negotiate difficulties in their usability and reliability and address concerns and worries regarding privacy loss (43).

### Communities of Patients

Social media offers an opportunity for persons living with diabetes to access expert peer advice about making care fit. There is scant evidence about the impact of interventions within social media networks on diabetes outcomes (44). A “netnographic” study across social media platforms showed that patients with diabetes gained access to patient experts who offered practical answers and problem-solving tips and hacks, received and offered emotional support, and developed capacity through an enriched sense of connection (45). A 2018 analysis found almost 200,000 persons living with diabetes participating in Facebook diabetes groups, most of which focused on practical problem solving (46). These findings are consistent with a recently published review (47), demonstrating that social media participation may contribute to improve patient capacity to fit care both by contributing to practical know-how and self-efficacy and by the emotional enrichment of taking part in a community of reciprocal relationships.

With the responsibility of self-management residing almost completely with the patient, and the technological advances over the last decades, the diabetes online community (DOC) is growing as an expert support system (48). The DOC is a widely used term, encompassing all people engaging in diabetes-related online activities, e.g., blogs, discussion and support groups, video tutorials, podcasts, and other offerings (49). With patients often reporting diabetes to exert a negative impact on their relationships and their physical health (50), diabetes medication to interfere with living a normal life (50), and having to deal with social stigma (51), support is crucial to reduce associated feelings of distress and burnout (52). With diabetes distress being highly prevalent in patients with diabetes and associated to poor diabetes self-management, reducing diabetes distress is essential to improve diabetes-related outcomes and improve quality of life (52, 53). People with chronic health conditions often endorse feeling more comfortable sharing their experiences and struggles with others who can relate based on their own lived experiences (54). Patients can feel more supported through digital contact with peers and fellow patients which in turn can contribute to improve self-care routines, effective problem-solving and lifestyle adjustments, quality of life, and outcomes of care (54–57). This work with online peers may be emotionally less “costly” than the support of more immediate family members, also critical as its absence has been associated with low treatment fidelity (58). The value of DOC may continue to increase with increases in the incidence of diabetes distress, high burden of disease and treatment, and social isolation. Fitting care at the point of life will become increasingly important with the advent of innovations such as new therapeutic agents, transplantation of the islets of Langerhans, the introduction of organoids, and the development of high-functioning artificial pancreas systems. Expert patients and DOC will play a central role in the cautious but opportune adoption of these advances, sharing their knowledge and experiences with different treatment options, supporting patients in the decision-making process to adopt new and experimental technologies, manage expectations, and facilitating their normalization within the routines of people’s lives. With the evolving role of patients becoming experts in their own medical situation with the help of online communities and social media, it is important for clinicians to adopt an open and supportive approach towards online support.

## Discussion

Patient empowerment and patient-centered care are essential for the optimal care of people living with type 1 or type 2 diabetes. Evidence-based diabetes care, to be person centered, demands that clinicians become skilled in supporting SDM and work toward MDM (12). Healthcare systems and policies can promote or hinder this approach, for example, by shifting attention, quality metrics, and financial incentives from technical healthcare outcomes like HbA1c toward more holistic outcomes such as quality of life and burden of treatment. While expert guidelines progress in this direction, and the patient community makes increasingly important contributions to care that fits, implementation currently still lags at the point of care as a result of HbA1c dependent reimbursements by insurance companies and insufficient awareness, knowledge, and practical guidance within clinical practice to improve the fit of care. We must work toward a reality in which each person with diabetes is seen not as an object, a diagnosis, a subject of treatment with a predetermined universal goal, but rather as a complex person within a problematic human situation and imbued of personal and community values for whom an evidence-based care plan must be co-created, crafted carefully to meet her needs and advance her priorities. Clinicians and patients working side-by-side as complimentary experts and partners in implementation, can form not just a plan of care that fits in the clinic, but a safe and effective one that fits well in patients’ daily lives.

## Author Contributions

MR, RR-G, VM, and MK wrote the manuscript together. MK did the final review of the manuscript. MR, RR-G, VM, and MK are all guarantors of this work and, as such, had access to all articles and take responsibility for the integrity of the article. All authors contributed to the article and approved the submitted version.

## Funding

MK has received a ZonMw Veni Award (016.196.138) to fund her research activities.

## Conflict of Interest

The authors declare that the research was conducted in the absence of any commercial or financial relationships that could be construed as a potential conflict of interest.

## References

[1] Tamhane S Rodriguez-Gutierrez R Hargraves I Montori VM. Shared Decision-Making in Diabetes Care. Curr Diabetes Rep (2015) 15(12):112. doi: 10.1007/s11892-015-0688-0 26458383

[2] Kunneman M Brito JP Montori VM. Making Diabetes Care Fit. In: Diabetes Update. Waltham, MA: NEJM Group (2020). p. 18.

[3] Montori VM Kunneman M Hargraves I Brito JP. Shared decision making and the internist. Eur J Internal Med (2017) 37:1–6. doi: 10.1016/j.ejim.2016.09.018 27712953

[4] American Diabetes A. 16. Diabetes Advocacy: Standards of Medical Care in Diabetes-2021. Diabetes Care (2021) 44(Suppl 1):S221–2. doi: 10.2337/dc21-S016 33298427

[5] Garber AJ Handelsman Y Grunberger G Einhorn D Abrahamson MJ Barzilay JI. Consensus Statement by the American Association of Clinical Endocrinologists and American College of Endocrinology on the Comprehensive Type 2 Diabetes Management Algorithm - 2020 Executive Summary. Endocr Pract (2020) 26(1):107–39. doi: 10.4158/CS-2019-0472 32022600

[6] Rodriguez-Gutierrez R Gonzalez-Gonzalez JG Zuniga-Hernandez JA McCoy RG. Benefits and harms of intensive glycemic control in patients with type 2 diabetes. BMJ (2019) 367:l5887. doi: 10.1136/bmj.l5887 31690574

[7] Boussageon R Bejan-Angoulvant T Saadatian-Elahi M Lafont S Bergeonneau C Kassai B. Effect of intensive glucose lowering treatment on all cause mortality, cardiovascular death, and microvascular events in type 2 diabetes: meta-analysis of randomised controlled trials. BMJ (2011) 343:d4169. doi: 10.1136/bmj.d4169 21791495 PMC3144314

[8] Hemmingsen B Lund SS Gluud C Vaag A Almdal T Hemmingsen C. Intensive glycaemic control for patients with type 2 diabetes: systematic review with meta-analysis and trial sequential analysis of randomised clinical trials. BMJ (2011) 343:d6898. doi: 10.1136/bmj.d6898 22115901 PMC3223424

[9] Rodriguez-Gutierrez R Lipska KJ McCoy RG Ospina NS Ting HH Montori VM. Hypoglycemia as an indicator of good diabetes care. BMJ (2016) 352:i1084. doi: 10.1136/bmj.i1084 26951142 PMC6886856

[10] Rodriguez-Gutierrez R Lipska KJ McCoy RG. Intensive Glycemic Control in Type 2 Diabetes Mellitus – A Balancing Act of Latent Benefit and Avoidable Harm: A Teachable Moment. JAMA Internal Med (2016) 176(3):300–1. doi: 10.1001/jamainternmed.2015.8320 PMC5050553 26882223

[11] Spencer-Bonilla G Quiñones AR Montori VM. International Minimally Disruptive Medicine W. Assessing the Burden of Treatment. J Gen Intern Med (2017) 32(10):1141–5. doi: 10.1007/s11606-017-4117-8 PMC5602768 28699060

[12] May C Montori VM Mair FS. We need minimally disruptive medicine. BMJ (2009) 339:b2803. doi: 10.1136/bmj.b2803 19671932

[13] Funnell MM Anderson RM Arnold MS Barr PA Donnelly M Johnson PD. Empowerment: an idea whose time has come in diabetes education. Diabetes Education (1991) 17(1):37–41. doi: 10.1177/014572179101700108 1986902

[14] Anderson RM Funnell MM. Patient empowerment: myths and misconceptions. Patient Educ Couns (2010) 79(3):277–82. doi: 10.1016/j.pec.2009.07.025 PMC2879465 19682830

[15] Chatterjee S Davies MJ Heller S Speight J Snoek FJ Khunti K. Diabetes structured self-management education programmes: a narrative review and current innovations. Lancet Diabetes Endocrinol (2018) 6(2):130–42. doi: 10.1016/S2213-8587(17)30239-5 28970034

[16] America Iomcoqohci. Crossing the quality chasm: A new health care system for the 21st century. Natl Acad Sci (2001). doi: 10.17226/10027

[17] Little P Everitt H Williamson I Warner G Moore M Gould C. Preferences of patients for patient centred approach to consultation in primary care: observational study. BMJ (2001) 322(7284):468–72. doi: 10.1136/bmj.322.7284.468 PMC26564 11222423

[18] Constand MK MacDermid JC Dal Bello-Haas V Law M. Scoping review of patient-centered care approaches in healthcare. BMC Health Serv Res (2014) 14(1):271. doi: 10.1186/1472-6963-14-271 24947822 PMC4079171

[19] Powers MA Bardsley JK Cypress M Funnell MM Harms D Hess-Fischl A. Diabetes Self-management Education and Support in Adults With Type 2 Diabetes: A Consensus Report of the American Diabetes Association, the Association of Diabetes Care & Education Specialists, the Academy of Nutrition and Dietetics, the American Academy of Family Physicians, the American Academy of PAs, the American Association of Nurse Practitioners, and the American Pharmacists Association. Diabetes Care (2020) 43(7):1636–49. doi: 10.2337/dci20-0023 32513817

[20] Kunneman M Montori VM Castaneda-Guarderas A Hess EP. What Is Shared Decision Making? (and What It Is Not). Acad Emergency Med (2016) 23(12):1320–4. doi: 10.1111/acem.13065 27770514

[21] Rodriguez-Gutierrez R Gionfriddo MR Singh Ospina N Maraka S Tamhane S Montori VM. Shared decision making in endocrinology: present and future directions. Lancet Diabetes Endocrinol (2016) 4(8):706–16. doi: 10.1016/S2213-8587(15)00468-4 26915314

[22] Charles C Gafni A Whelan T. Decision-making in the physician–patient encounter: revisiting the shared treatment decision-making model. Soc Sci Med (1999) 49(5):651–61. doi: 10.1016/S0277-9536(99)00145-8 10452420

[23] Montori VM Gafni A Charles C. A shared treatment decision-making approach between patients with chronic conditions and their clinicians: the case of diabetes. Health Expect (2006) 9(1):25–36. doi: 10.1111/j.1369-7625.2006.00359.x 16436159 PMC5060323

[24] Stacey D Légaré F Lewis K Barry MJ Bennett CL Eden KB. Decision aids for people facing health treatment or screening decisions. Cochrane Database Systematic Rev (2017) 4(4):CD001431. doi: 10.1002/14651858.CD001431.pub5 PMC6478132 28402085

[25] Barry MJ Edgman-Levitan S. Shared Decision Making — The Pinnacle of Patient-Centered Care. New Engl J Med (2012) 366(9):780–1. doi: 10.1056/NEJMp1109283 22375967

[26] Rabi DM Kunneman M Montori VM. When Guidelines Recommend Shared Decision-making. JAMA (2020) 323(14):1345–6. doi: 10.1001/jama.2020.1525 32167526

[27] Zeballos-Palacios CL Hargraves IG Noseworthy PA Branda ME Kunneman M Burnett B. Developing a Conversation Aid to Support Shared Decision Making: Reflections on Designing Anticoagulation Choice. Mayo Clin Proc (2019) 94(4):686–96. doi: 10.1016/j.mayocp.2018.08.030 PMC6450705 30642640

[28] Hargraves I Montori VM. Decision aids, empowerment, and shared decision making. BMJ (2014) 349:g5811. doi: 10.1136/bmj.g5811 25255800

[29] https://diabetesdecisionaid.mayoclinic.org/index.

[30] Maiorino MI Signoriello S Maio A Chiodini P Bellastella G Scappaticcio L. Effects of Continuous Glucose Monitoring on Metrics of Glycemic Control in Diabetes: A Systematic Review With Meta-analysis of Randomized Controlled Trials. Diabetes Care (2020) 43(5):1146–56. doi: 10.2337/dc19-1459 32312858

[31] Weisman A Bai JW Cardinez M Kramer CK Perkins BA. Effect of artificial pancreas systems on glycaemic control in patients with type 1 diabetes: a systematic review and meta-analysis of outpatient randomised controlled trials. Lancet Diabetes Endocrinol (2017) 5(7):501–12. doi: 10.1016/S2213-8587(17)30167-5 28533136

[32] Barnard KD Lloyd CE Skinner TC. Systematic literature review: quality of life associated with insulin pump use in Type 1 diabetes. Diabetes Med (2007) 24(6):607–17. doi: 10.1111/j.1464-5491.2007.02120.x 17367304

[33] Jeitler K Horvath K Berghold A Gratzer TW Neeser K Pieber TR. Continuous subcutaneous insulin infusion versus multiple daily insulin injections in patients with diabetes mellitus: systematic review and meta-analysis. Diabetologia (2008) 51(6):941–51. doi: 10.1007/s00125-008-0974-3 18351320

[34] Messer LH Cook PF Tanenbaum ML Hanes S Driscoll KA Hood KK. CGM Benefits and Burdens: Two Brief Measures of Continuous Glucose Monitoring. J Diabetes Sci Technol (2019) 13(6):1135–41. doi: 10.1177/1932296819832909 PMC6835174 30854886

[35] https://carethatfits.org/my-life-my-healthcare/.

[36] Arnold SRC Riches VC Stancliffe RJ. I-CAN. The Classification and Prediction of Support Needs. J Appl Res Intellectual Disabilities (2014) 27(2):97–111. doi: 10.1111/jar.12055 23666847

[37] Warshaw H Isaacs D MacLeod J. The Reference Guide to Integrate Smart Insulin Pens Into Data-Driven Diabetes Care and Education Services. Diabetes Educator (2020) 46(4_suppl):3S–20S. doi: 10.1177/0145721720930183 32779975

[38] Brown SA Breton MD Anderson SM Kollar L Keith-Hynes P Levy CJ. Overnight Closed-Loop Control Improves Glycemic Control in a Multicenter Study of Adults With Type 1 Diabetes. J Clin Endocrinol Metab (2017) 102(10):3674–82. doi: 10.1210/jc.2017-00556 PMC5630248 28666360

[39] Yaron M Roitman E Aharon-Hananel G Landau Z Ganz T Yanuv I. Effect of Flash Glucose Monitoring Technology on Glycemic Control and Treatment Satisfaction in Patients With Type 2 Diabetes. Diabetes Care (2019) 42(7):1178–84. doi: 10.2337/dc18-0166 31036546

[40] Wada E Onoue T Kobayashi T Handa T Hayase A Ito M. Flash glucose monitoring helps achieve better glycemic control than conventional self-monitoring of blood glucose in non-insulin-treated type 2 diabetes: a randomized controlled trial. BMJ Open Diabetes Res Care (2020) 8(1):e001115. doi: 10.1136/bmjdrc-2019-001115 PMC7292039 32518063

[41] Al Hayek AA Robert AA Al Dawish MA. Evaluation of FreeStyle Libre Flash Glucose Monitoring System on Glycemic Control, Health-Related Quality of Life, and Fear of Hypoglycemia in Patients with Type 1 Diabetes. Clin Med Insights: Endocrinol Diabetes (2017) 10:1–6. doi: 10.1177/1179551417746957 PMC5731614 29270042

[42] Peyrot M Rubin RR. Patient-reported outcomes for an integrated real-time continuous glucose monitoring/insulin pump system. Diabetes Technol Ther (2009) 11(1):57–62. doi: 10.1089/dia.2008.0002 19132857

[43] Grundy Q Chiu K Held F Continella A Bero L Holz R. Data sharing practices of medicines related apps and the mobile ecosystem: traffic, content, and network analysis. BMJ (2019) 364:l920. doi: 10.1136/bmj.l920 30894349 PMC6425456

[44] Gabarron E Arsand E Wynn R. Social Media Use in Interventions for Diabetes: Rapid Evidence-Based Review. J Med Internet Res (2018) 20(8):e10303. doi: 10.2196/10303 30097421 PMC6109225

[45] Tenderich A Tenderich B Barton T Richards SE. What Are PWDs (People With Diabetes) Doing Online? A Netnographic Analysis. J Diabetes Sci Technol (2019) 13(2):187–97. doi: 10.1177/1932296818813192 PMC6399800 30477335

[46] Stellefson M Paige S Apperson A Spratt S. Social Media Content Analysis of Public Diabetes Facebook Groups. J Diabetes Sci Technol (2019) 13(3):428–38. doi: 10.1177/1932296819839099 PMC6501525 30931593

[47] Oser TK Oser SM Parascando JA Hessler-Jones D Sciamanna CN Sparling K. Social Media in the Diabetes Community: a Novel Way to Assess Psychosocial Needs in People with Diabetes and Their Caregivers. Curr Diabetes Rep (2020) 20(3):10. doi: 10.1007/s11892-020-1294-3 32080765

[48] Hilliard ME Sparling KM Hitchcock J Oser TK Hood KK. The emerging diabetes online community. Curr Diabetes Rev (2015) 11(4):261–72. doi: 10.2174/1573399811666150421123448 PMC4586085 25901500

[49] Litchman ML Walker HR Ng AH Wawrzynski SE Oser SM Greenwood DA. State of the Science: A Scoping Review and Gap Analysis of Diabetes Online Communities. J Diabetes Sci Technol (2019) 13(3):466–92. doi: 10.1177/1932296819831042 PMC6501517 30854884

[50] Nicolucci A Kovacs Burns K Holt RIG Comaschi M Hermanns N Ishii H. Diabetes Attitudes, Wishes and Needs second study (DAWN2™): Cross-national benchmarking of diabetes-related psychosocial outcomes for people with diabetes. Diabetic Med (2013) 30(7):767–77. doi: 10.1111/dme.12245 23711019

[51] Liu NF Brown AS Folias AE Younge MF Guzman SJ Close KL. Stigma in People With Type 1 or Type 2 Diabetes. Clin. Diabetes (2017) 35(1):27–34. doi: 10.2337/cd16-0020 28144043 PMC5241772

[52] Fisher L Mullan JT Arean P Glasgow RE Hessler D Masharani U. Diabetes distress but not clinical depression or depressive symptoms is associated with glycemic control in both cross-sectional and longitudinal analyses. Diabetes Care (2010) 33(1):23–8. doi: 10.2337/dc09-1238 PMC2797978 19837786

[53] Gonzalez JS Fisher L Polonsky WH. Depression in diabetes: have we been missing something important? Diabetes Care (2011) 34(1):236–9. doi: 10.2337/dc10-1970 PMC3005471 21193621

[54] Dale JR Williams SM Bowyer V. What is the effect of peer support on diabetes outcomes in adults? A systematic review. Diabetic Med (2012) 29(11):1361–77. doi: 10.1111/j.1464-5491.2012.03749.x 22804713

[55] Kingod N Cleal B Wahlberg A Husted GR. Online Peer-to-Peer Communities in the Daily Lives of People With Chronic Illness:A Qualitative Systematic Review. Qual. Health Res (2017) 27(1):89–99. doi: 10.1177/1049732316680203 27956659

[56] Litchman ML Edelman LS Donaldson GW. Effect of Diabetes Online Community Engagement on Health Indicators: Cross-Sectional Study. JMIR Diabetes (2018) 3(2):e8. doi: 10.2196/diabetes.8603 30291079 PMC6238850

[57] Greenhalgh T Collard A Begum N. Sharing stories: complex intervention for diabetes education in minority ethnic groups who do not speak English. BMJ (2005) 330(7492):628. doi: 10.1136/bmj.330.7492.628 15774990 PMC554907

[58] Mayberry LS Osborn CY. Family support, medication adherence, and glycemic control among adults with type 2 diabetes. Diabetes Care (2012) 35(6):1239–45. doi: 10.2337/dc11-2103 PMC3357235 22538012

